# Alginate Ag/AgCl Nanoparticles Composite Films for Wound Dressings with Antibiofilm and Antimicrobial Activities

**DOI:** 10.3390/jfb14020084

**Published:** 2023-02-01

**Authors:** Matteo Puccetti, Anna Donnadio, Maurizio Ricci, Loredana Latterini, Giulia Quaglia, Donatella Pietrella, Alessandro Di Michele, Valeria Ambrogi

**Affiliations:** 1 Dipartimento di Scienze Farmaceutiche, Università degli Studi di Perugia, Via del Liceo 1, 06123 Perugia, Italy; 2 Nano4Light Lab, Dipartimento di Chimica, Biologia e Biotecnologie, Università degli Studi di Perugia, Via Elce di Sotto, 8, 06123 Perugia, Italy; 3 Dipartimento di Medicina e Chirurgia, Università degli Studi di Perugia, Via Piazzale Gambuli, 1, 06129 Perugia, Italy; 4 Dipartimento di Fisica e Geologia, Università degli Studi di Perugia, Via Pascoli, 06123 Perugia, Italy

**Keywords:** Ag nanoparticles, Ag/Cl nanoparticles, calcium alginate films, antibacterial and antifungal activities, cytotoxicity

## Abstract

Recently, silver-based nanoparticles have been proposed as components of wound dressings due to their antimicrobial activity. Unfortunately, they are cytotoxic for keratinocytes and fibroblasts, and this limits their use. Less consideration has been given to the use of AgCl nanoparticles in wound dressings. In this paper, a sustainable preparation of alginate AgCl nanoparticles composite films by simultaneous alginate gelation and AgCl nanoparticle formation in the presence of CaCl_2_ solution is proposed with the aim of obtaining films with antimicrobial and antibiofilm activities and low cytotoxicity. First, AgNO_3_ alginate films were prepared, and then, gelation and nanoparticle formation were induced by film immersion in CaCl_2_ solution. Films characterization revealed the presence of both AgCl and metallic silver nanoparticles, which resulted as quite homogeneously distributed, and good hydration properties. Finally, films were tested for their antimicrobial and antibiofilm activities against *Staphylococcus epidermidis* (ATCC 12228), *Staphylococcus aureus* (ATCC 29213), *Pseudomonas aeruginosa* (ATCC 15692), and the yeast *Candida albicans*. Composite films showed antibacterial and antibiofilm activities against the tested bacteria and resulted as less active towards *Candida albicans*. Film cytotoxicity was investigated towards human dermis fibroblasts (HuDe) and human skin keratinocytes (NCTC2544). Composite films showed low cytotoxicity, especially towards fibroblasts. Thus, the proposed sustainable approach allows to obtain composite films of Ag/AgCl alginate nanoparticles capable of preventing the onset of infections without showing high cytotoxicity for tissue cells.

## 1. Introduction

Hard-to-heal wounds heal slowly, have a high recurrence rate, and are associated with the presence of infection and copious exudate. The presence of microorganisms in wounds has been recognized as a significant cause for healing delay. Moreover, chronic wounds are often complicated by the presence of biofilms, inflammation, and/or non-viable tissue production. Non-healing wounds, as an example, are among the serious complications of type 2 diabetes in the world, associated with a high incidence of bacterial infections, impaired immunity, and other complications that can lead to limb amputation [1]. Current therapeutic approaches are, therefore, multifaceted and focused on a rapid healing as well as recurrence prevention. Advanced wound dressings, especially absorbent ones with improved performance, obtained with sustainable methods, and at the same time not based on the use of antibiotics, are an important aspect of wound care in many clinical settings.

Silver has been used in wound care for a long time [2], and it remains a popular active agent for wound treatment still today. It is available in numerous forms and has a broad spectrum of activity. Newer strategies in using silver for wound healing should be oriented towards prolonged release formulations to obtain local drug concentrations able to explain the antimicrobial activity without resulting in local tissue damage. Recently, with advancements in nanotechnology, silver metallic nanoparticles have been attracting the interest of researchers, and their use in wound healing has been proposed because of their unique antimicrobial properties [3,4,5,6,7,8]. Less attention has been paid to the use of AgCl nanoparticles in wound dressing although their antimicrobial activity is largely known [9,10,11,12,13,14,15,16], and to the best of our knowledge, this topic has not been largely investigated [17,18,19]. Alginate has long been used in a variety of biomedical applications owing to its many favorable properties including biocompatibility and low toxicity [20]. Of note, alginate can be tailored as a material with properties suitable for wound healing such as the ability to absorb excess exudates, maintaining a physiologically moist environment, and minimizing wound bacterial infections. Moreover, alginate hydrogels can incorporate silver nanoparticles to obtain wound dressings joining both the previously mentioned polymer characteristics with the silver antimicrobial effects [21,22]. Alginate films containing silver nanoparticles supported on pyrogenic silica have been described as potentially innovative wound dressings characterized by antimicrobial activity and lack of cytotoxic effects against fibroblasts and keratinocytes [6]. Some silver-based alginate wound dressings are already present in the market [23,24]. In these dressings, silver is present in ionic form, as a salt such as AgNO_3_ or as silver sodium hydrogen zirconium phosphate, silver sulphadiazine, or in metallic silver nanocrystals.

Alginate sodium is an anionic biopolymer obtained from brown seaweed. It is a linear water-soluble block co-polymer whose monomers are β-D-mannuronic and α-L-guluronic acids in different proportions, bound by 1,4-links. When treated with solutions of divalent cations, such as calcium ions, it undergoes fast gelation. These ions act as crosslinkers that interact between guluronic acid (G)-rich regions of adjacent chains, giving rise to the formation of a bulk “egg-box” structure [25].

Recently, an easy and ecofriendly synthetic approach for the preparation of calcium alginate/silver chloride nanocomposite has been reported [15] following the in situ synthesis of silver chloride nanoparticles in alginate dispersion. The composite was tested for antibacterial activity and proposed as a promising candidate for applications in the textile industry as well as a slow-smoke flame retardant.

In the present paper, a modified procedure is proposed to obtain wound dressings with antimicrobial activity. It consists of the immersion of previously prepared AgNO_3_ loaded alginate films in a CaCl_2_ solution, thus inducing in one step both the alginate gelation and the Ag/Cl nanoparticles formation. In comparison to the above-described method [15], the present procedure does not include the presence of ammonia, which is often used as a silver-stabilizing agent, and thus, no other reagents were used in addition to those present in the final films. Glycerin was added as a plasticizer agent. The obtained films were characterized with respect to nanoparticle nature, hydration properties, and silver release. Moreover, the antimicrobial and cytotoxic activities were evaluated and compared to those of AgNO_3_-loaded non-crosslinked alginate films.

## 2. Materials and Methods

### 2.1. Materials

Sodium alginate was bought from Sigma-Aldrich Chemical (Milan, Italy, cod.180947), glycerol 85% from Acef (Fiorenzuola d’Adda, Italy), silver nitrate 99+% from Alfa Aesar (Karlsruhe, Germany), and calcium chloride from Sigma-Aldrich Chemical (Milan, Italy, cod.C1016). Deionized water obtained via a reverse-osmosis procedure (MilliQ system, Millipore, Rome, Italy) was used. Other reagents and solvents were from Sigma-Aldrich and were used as received.

### 2.2. Characterization

X-ray powder diffraction (XRPD) patterns were obtained with the Cu-Kα radiation on a Bruker D8 Advance diffractometer and PW3050 goniometer provided with a Lynxeye detector. The long fine-focus (LFF) ceramic tube was operated at 40 kV and 40 mA. DIFFRAC.EVA V5 software was used for the phase identification (software version 2.0 up, © 2010–2019 Bruker AXS GmbH, Karlsruhe, Germany) and was equipped with COD database.

Particle morphology, surface characteristics, and particle aggregation were examined using an FEG LEO 1525 scanning electron microscope (LEO Electron Microscopy Inc., NY) with an acceleration potential voltage of 1 keV. Samples were placed onto carbon tape-coated aluminum stubs and, before imaging, were sputter-coated with chromium by a high-resolution sputter (Quorum Technologies, East Essex, UK) at 20 mA for 20 s.

A Perkin-Elmer Lambda 800 double-beam UV–vis spectrometer was used to record the absorption spectra and was equipped with a data processor for recording and displaying spectra. 

Reflectance spectra of the powder samples were recorded by a Varian (Cary 4000) spectrophotometer that was equipped with a 150 nm integrating sphere (DRA-900). 

The light reflected from the sample surface in all directions was collected from the sphere and directed towards the photomultiplier. A fully reflective barium sulfate plate was used as a reference. The connected detector was a photomultiplier. 

Quantitative Ag determination was performed with a Varian 700-ES series inductively coupled plasma-optical emission spectrometer (ICP-OES) with axial injection.

### 2.3. Film Preparation 

Alginate film was prepared by dispersing sodium alginate (2% *w*/*w*) in water, then adding glycerin (1% *w*/*w*), and leaving the dispersion under magnetic stirring at room temperature for about 12 h until a homogeneous dispersion was obtained. Then, air bubbles were removed using the THINKY ARE-250 at 2000 rpm for 20 min. Finally, the dispersion (25 g) was poured into a 9 cm diameter Petri dish and then dried in a desiccator in the presence of P_2_O_5_ and under N_2_ flow for 7–8 h. The film will be later referred to as film β0. Once dried, film β0 was immersed in 1.6% (*w*/*w*) CaCl_2_ aqueous solution in the dark for 24 h. Then, the gelled film was washed for three times with deionized water. Successively, the excess water was dabbed with a filter paper, and then, the film was maintained in the presence of magnesium nitrate (RH 53%) in a desiccator to give the film referred to as film β0CaCl_2_.

Sodium alginate composite films containing different amounts of AgNO_3_ (Table 1) were obtained following the procedure described above. Finally, the obtained composite films were gelled by immersion in 1.6% (*w*/*w*) CaCl_2_ aqueous solution to give rise to cross-linked films and nanoparticles formation.

### 2.4. Swelling Test

Quadrate samples (2 mm × 2 mm) were cut from the films β0CaCl_2_, β1CaCl_2_, and β2CaCl_2_. They were weighed (W_1_) and sunk into a simulated wound fluid composed of an aqueous solution of 0.4 M sodium chloride, 0.02 M calcium chloride, and 0.08 M tris (hydroxymethyl)amino-methane without bovine serum albumin (pH 7.5) [26]. The test was performed at 37 °C.

At set times (0.5, 1, 2, 4, 6, and 24 h), the samples were taken, dabbed with filter paper, and weighed (W_2_). The following formula was used to measure the water uptake expressed as % of hydration:

% of hydration = [(W_2_ − W_1_)/W_1_] × 100 (1)

For the determination of dissolution degree, the films immersed for 24 h were weighed and dried at 40 °C for 24 h and maintained in a desiccator for 48 h. Finally, they were weighed again (W_3_). The dissolution degree (DD) was determined by the following formula:

DD = [(W_1_ − W_3_)/W_1_] × 100% (2)

This test was executed three times, and results were expressed as an average.

### 2.5. In Vitro Silver Release 

Circular films (2 cm diameter) were immersed in 10 mL of simulated wound fluid at 37 ± 0.5 °C with stirring (80 rpm). At set times, withdrawals (1 mL) were done and immediately replenished by the neat preheated fluid (1 mL). The sample was properly diluted with conc. HNO_3_ and deionized water, and the silver content was measured by using an ICP-OES. The results were expressed as an average of three experiments.

### 2.6. Microorganisms

*Staphylococcus aureus* (ATCC 29213) and *Staphylococcus epidermidis* (ATCC 12228) as Gram-positive bacteria, *Pseudomonas aeruginosa* (ATCC 15692) as the Gram-negative microorganism, and the yeast *Candida albicans* (SC5314) were used as microbial strains for evaluating the antimicrobial activity of the films described in this study. Bacteria were grown in Muller Hinton agar (MHA), whereas for *Candida* cells in Sabouraud agar. Oone colony of each microorganism was inoculated in the appropriate culture broth medium (Muller Hinton broth MHB or Sabouraud broth) and maintained for 24 h at 37 °C. Then, after centrifugation, the recovered microorganisms were washed twice in sterile saline and counted by spectrophotometric analysis. The suspensions were then diluted in the appropriate culture medium to the concentration required in the following assays.

### 2.7. Antimicrobial Activity

To determine the antimicrobial activity, the Kirby–Bauer disk diffusion test was performed, and the Clinical Laboratory Standards directions were followed. The zone of inhibition (ZOI) was evaluated according to the method previously described [27] with some modifications. Microorganism suspensions were washed in sterile phosphate buffer, and cell concentration was measured by spectrophotometry (600 nm). Microbial cells (1 × 10^8^ per mL) were uniformly sown on MHA or SDA plates. Film samples (6 mm diameter disks) were sterilized by UV and placed on the surface of the MHA or SDA plates. Then, disks were hydrated with 20 μL sterile water and finally incubated (37 °C, 24 h). Film β0CaCl_2_ was used as a negative control, whereas the positive control was constituted by disks containing gentamicin (30 µg). ZOI was calculated by measuring, with a metric ruler, the diameter of the clear zone around the disks. Data were expressed in millimeters (mm) as mean ± standard deviation (SD) of separated experiments (*n* = 3). 

### 2.8. Antibiofilm Activity

The antibiofilm activity was performed in an in vitro model of static biofilm assay as previously described and properly modified [28]. The test was conducted in a 96-well microtiter plate. Bacteria were seeded on MHB medium with *Candida albicans* in Sabouraud broth and grown overnight at 37 °C. Then, the organisms are diluted in 2% sucrose MHB until a concentration of 10^6^–10^7^ cells/mL was obtained. Using a 96-well flat-bottomed polystyrene plate, 200 µL of suspension were inoculated in the presence of alginate-based films. Gentamicin (2.5 μg/mL) and fluconazole (5 µg/mL) were used as positive controls for bacteria and *Candida albicans,* respectively. After incubation for 24 h at 37 °C, biofilms in each well were washed twice with sterile water (200 μL) and dried for 45 min. Staining was carried on by adding 0.4% crystal violet (100 μL) to each well for 30–45 min. Biofilms were then washed with water for four times, and crystal violet was dissolved in 95% ethanol (200 μL) for 45 min. Absorbance of crystal violet (570 nm) was measured on a microplate reader (Tecan). The antibiofilm activity was calculated by comparison of the absorbance values of the biofilm obtained after treatment with film versus control biofilms. The results were expressed as mean ± SD of the absorbance values of two individual experiments performed in triplicate (*n* = 6). 

### 2.9. Cytotoxicity

To determine cytotoxicity, the cell ATP level was measured by ViaLight^®^ Plus Kit (Lonza, Milan, Italy). This specific kit allows the bioluminescence measurement of the amount of ATP present in metabolically active cells. Human dermis fibroblasts (HuDe) and human skin keratinocytes (NCTC2544) were used to test cytotoxicity of sterilized UV light films. The cells were grown to confluence in RPMI 1640 medium supplemented with streptomycin 10 μg/mL, 10,000 units penicillin, and 10% heat-inactivated fetal calf serum for 18 h. 

Monolayer cells were treated with films for 24 h at 37 °C, and then, plates were allowed to cool at room temperature for 10 min. Successively, the cell lysis reagent was added to each well to extract ATP from the cells and allowed to act for 15 min. Finally, the AMR Plus (ATP Monitoring Reagent Plus) was added, and after 2 more min, the luminescence was read by a TECAN microplate luminometer. Results are expressed as relative light units (RLU). 

## 3. Results and Discussion

### 3.1. Film Preparation and Characterization

**Figure 1:**
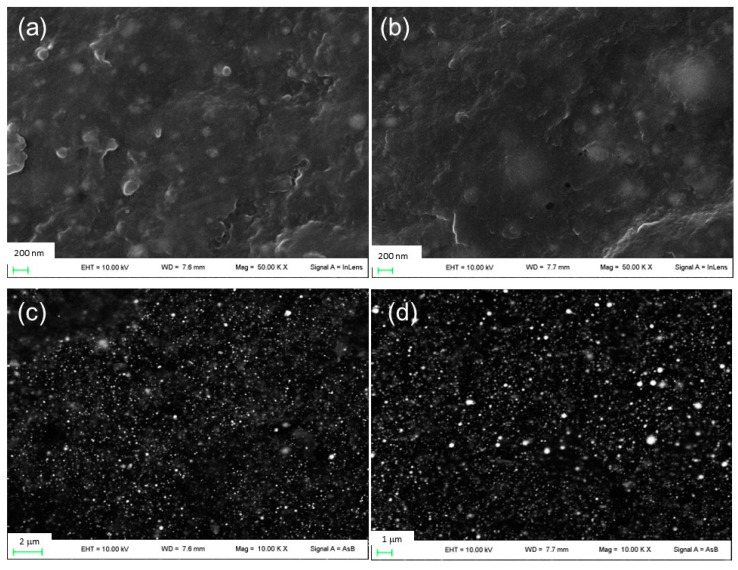
FE-SEM images of (**a**) film β1CaCl_2_ and (**b**) film β2CaCl_2_ and images of (**c**) film β1CaCl_2_ and (**d**) film β2CaCl_2_ obtained using backscattered electron detector.

Initially, sodium alginate films containing silver nitrate (film β1 and film β2) and neat sodium alginate (film β0) were prepared (Table 1, not-gelled films). The preparation of these sodium alginate films was performed by adding glycerin as a plasticizer agent. Glycerin, in fact, is reported to act as a plasticizer for improving alginate films physical properties [29,30,31]. These films were prepared according to our previous experience, which, based on macroscopic observations of flexibility and smoothness, suggested the proper alginate/glycerin ratio was 2/1 [6]. Then, the obtained films β0, β1, and β2 were dipped in a CaCl_2_ solution able to simultaneously induce alginate gelation by the divalent cation Ca^2+^ and the formation of AgCl nanoparticles by the presence of chloride anions. Thus, to determine the best CaCl_2_ concentration able to induce the formation of homogeneous and uniformly distributed nanoparticles, sodium alginate films were immersed in different CaCl_2_ solutions (0.8%, 1.2%, and 1.6 *w*/*w*). As observed in SEM micrographs (Figure S1), films prepared by dipping in a 0.8% CaCl_2_ solution showed the presence of bulky AgCl nanoparticles. Similar results were obtained when a 1.2% CaCl_2_ solution was used. By immersion in 1.6% *w*/*w* CaCl_2_ solution, films containing small and fairly uniform distributed nanoparticles were obtained, as shown in Figure 1. For comparison, a micrograph of film β0CaCl_2_ is reported in Figure S2. It can be noted that most of the inorganic nanoparticles are less than 100 nm in size. It can be supposed that the higher concentration of chloride anions in 1.6% CaCl_2_ solution allows a rapid and efficient AgCl nucleation limiting the crystal growth. Moreover, a higher Ca^2+^ concentration inducing a faster gelation of alginate [32] can reduce the diffusion of silver ions and consequent AgCl particle growth. In Table 1, the thickness of films before and after gelation is reported, and an increase of film thickness can be observed following the immersion in CaCl_2_ as reported by Russo et al. and Li et al. [32,33]. This can be due to the water absorption during crosslinking and to the stabilization of the conformation of the swollen state following gelation. In all films, the presence of silver caused a decrease in film thickness. Probably, silver-based nanoparticles induced a water absorption decrease (as proved hereafter), and thus, a minor swollen state was obtained.

**Figure 2:**
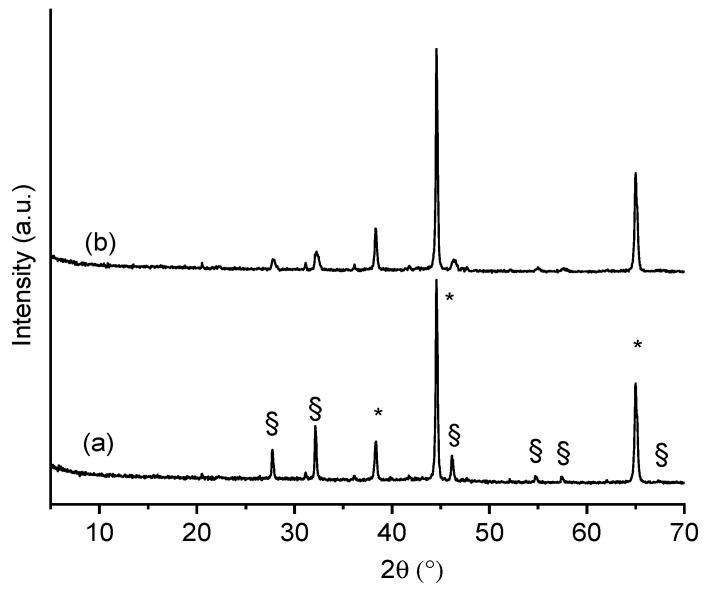
XRPD of (**a**) film β1CaCl_2_ and (**b**) film β2CaCl_2_. (*) Peaks ascribable to Ag nanoparticles; (§) peaks ascribable to AgCl nanoparticles.

The XRD patterns of films β1CaCl_2_ and β2CaCl_2_ are shown in Figure 2. It is possible to observe, beyond the crystallization of AgCl salt, the presence of metallic Ag nanoparticles. In particular, XRD patterns showed main peaks at 2θ: 27.8°, 32.2°, 46.3°, 54.70°, and 57.6°, which are characteristic of AgCl (COD number: 9011666), whereas peaks at 38.3°, 44.2°, and 65.1°, which corresponded to (111), (200), and (220) planes of face-centered cubic (fcc) crystal structure of metallic silver (COD number: 9011608), indicate the presence of Ag nanoparticles [34]. 

The differences in the peak intensities are due to the different amounts of silver in the final alginate films. The dimensions of AgCl nanoparticles were determined by applying the Scherrer equation to (111) and (200) and Bragg reflections at 2θ° 27.8 and 32.2 of the AgCl cubic phase, considering the correction for the instrumental line width broadening. The X-ray diffractograms were fitted by using a pseudo-Voigt profile function to obtain the FWHM related to (111) and (200) peaks. Based on this calculation, silver chloride nanoparticles obtained in β1CaCl_2_ and β2CaCl_2_ have a size of 65 and 25 nm, respectively. The dimensions of AgCl nanoparticles as from Scherrer equation were confronted with those showing the SEM images, and as can be noted, the nanoparticles sizes were larger than those estimated by XRD and distributed in a large dimensional range. 

**Figure 3:**
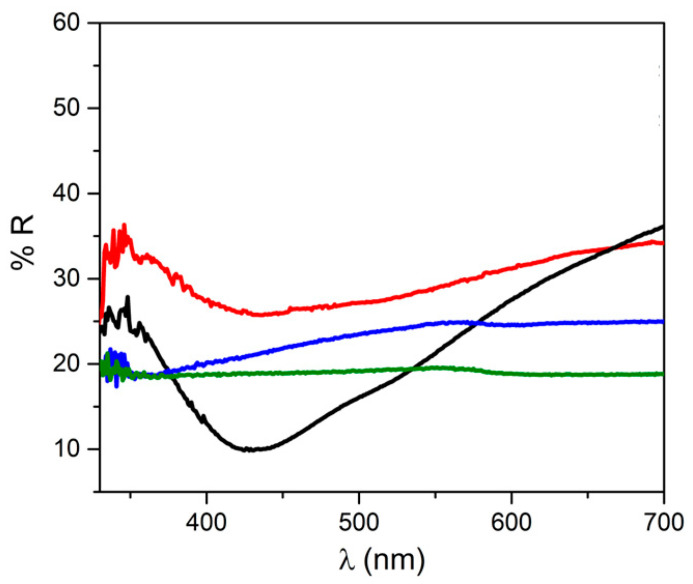
UV–vis reflectance spectra of film β0 (green line), β0CaCl_2_ (blue line), β1CaCl_2_ (black line), and β2CaCl_2_ (red line).

Figure 3 show the UV–vis reflectance spectra of films β1CaCl_2_ and β2CaCl_2_ together with those recorded on β0 and β0CaCl_2_ samples for comparison. Both spectra of the samples loaded with AgNO_3_ had a band centered on the 420–430 nm range, which is absent in the silver-free films (β0 and β0CaCl_2_); this band is due to the Ag nanoparticles surface plasmon resonance [9,35]. In both cases, the bands present an asymmetric profile likely due to the formation of an Ag-AgCl nanostructure [7].

From XRPD and UV–vis analyses, the presence of Ag/AgCl nanoparticles was proven, and thus, it can be asserted that nanoparticles can be obtained even in the absence of ammonia [15] and that the alginate gelation, with consequent reduction of alginate chain mobility, prevented agglomeration of AgCl nanoparticles.

### 3.2. Water Absorption

**Figure 4:**
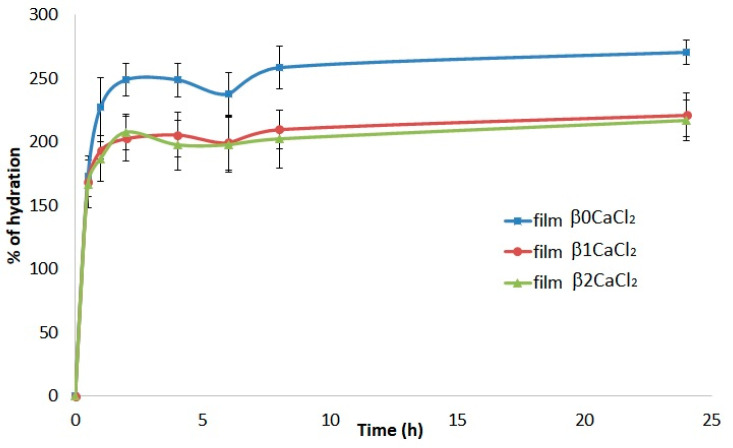
Percentage of hydration of the films β0CaCl_2_, β1CaCl_2_, and β2CaCl_2_ as a function of time.

Water absorption capacity is an important property of a wound-dressing material. In fact, good hydration properties allow the absorption of exudates, preventing maceration and providing a moist environment [36]. As shown in Figure 4, all films exhibited good and rapid hydration, and the highest absorption percentage was reached within 2 h. The percentage of hydration was slightly higher for the control film β0CaCl_2_ (consisting of neat calcium alginate), and the presence of silver induced only a low decrease of hydration, with no differences between film β1CaCl_2_ and β2CaCl_2_. 

After drying (24 h at 40 °C), the films were weighed to assess the weight loss. In all cases, the weight-loss percentage was very low (15.8 ± 2% for film β0CaCl_2_, 18.2 ± 1.7% film β1CaCl_2_, and 17.1 ± 2.3% film β2CaCl_2_), indicating that all films after 24 h of immersion in simulated wound fluid almost completely maintained their integrity and that the presence of silver did not affect the film dissolution.

### 3.3. Antimicrobial Activity

**Figure 5:**
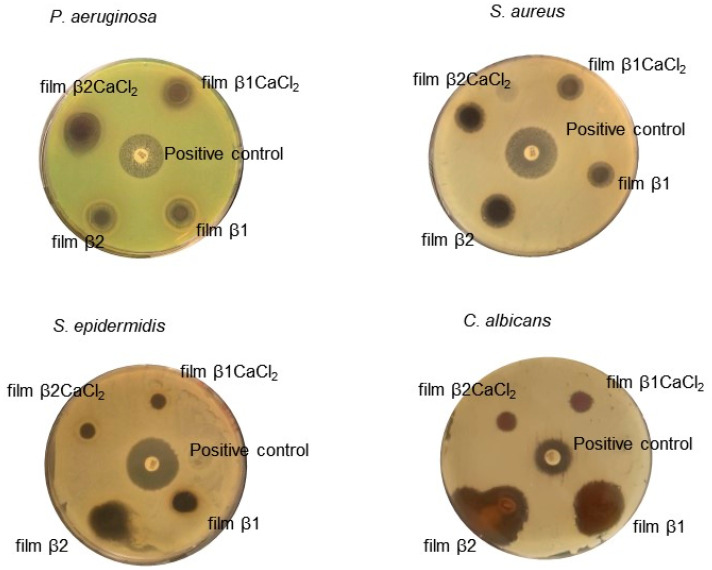
The Kirby–Bauer assay results. Paper disk soaked with 20 μg of gentamicin or fluconazole were used as a positive control. The results reported in the picture are representative of one of the three independent experiments.

Films antimicrobial activity was assessed by the Kirby–Bauer test. Film β0CaCl_2_ showed no antimicrobial activity, whereas un-gelled films β1 and β2 showed antimicrobial activity against all tested microorganisms (Figure 5). Unfortunately, it was not possible to measure the inhibition halos because these films in contact with agar medium dissolved, and the halo edges were enlarged. Films β1CaCl_2_ and β2CaCl_2_ showed antimicrobial activity against bacteria *Pseudomonas aeruginosa* and *Staphylococcus aureus* with the inhibition halos reported in Table 2, whereas in the case of *Staphylococcus epidermidis,* they showed antimicrobial activity in the contact areas of films with microorganisms. They showed low activity towards the yeast *Candida albicans*.

The antimicrobial mechanism of Ag/AgCl nanoparticles remains still unclear, and even if it is supposed that they exert their action with a mechanism similar to silver ions, some differences can be considered [37,38]. It has been hypothesized that nanoparticles can anchor to the bacterial cell and can be internalized. Moreover, they release silver ions, which bind molecules essential for microorganism survival, such as proteins, lipids, and DNA. In addition, Ag/AgCl nanoparticles promote the generation of reactive oxygen species, which are considered to be the main products leading to bacterial apoptosis [15,19].

### 3.4. Antibiofilm Activity

Since many bacteria grow in the form of biofilms, especially in chronic wounds, the antibiofilm activity of the prepared films against *P. aeruginosa, S. aureus, S. epidermidis,* and *C. albicans* was evaluated. Biofilms were grown in static conditions in the presence of composite films. The biofilm formation was evaluated by measuring the mass of the biofilm using crystal violet staining. For the un-gelled films, it was not possible to determine the growth of the biofilm, as they rapidly solved in the culture medium.

**Figure 6:**
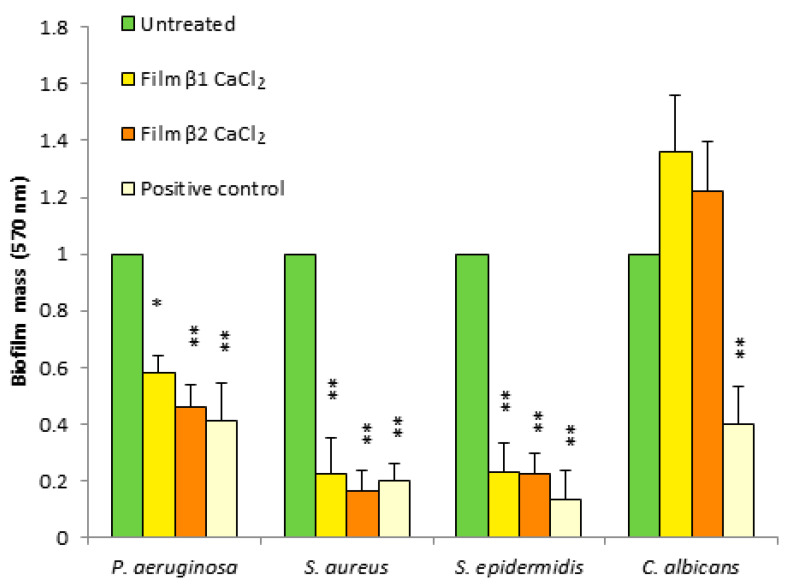
Antibiofilm activity of films β1CaCl_2_ and β2CaCl_2_ against *P. aeruginosa, S. aureus, S. epidermis*, and *C. albicans.* Untreated cells were considered as negative controls, and gentamicin- or fluconazole-treated cells were positive controls. Data are the mean ± SD of experiments carried out in triplicate. Values were normalized with respect to untreated cells considered 1. * *p* < 0.05, ** *p* < 0.01 (treated microorganisms versus untreated cells).

As shown in Figure 6, all films caused a reduction of mass biofilm against all tested bacteria, showing good antibiofilm activity against Gram-negative and Gram-positive bacteria. Instead, the films showed no effect on the formation of the *Candida albicans* yeast biofilm.

**Figure 7:**
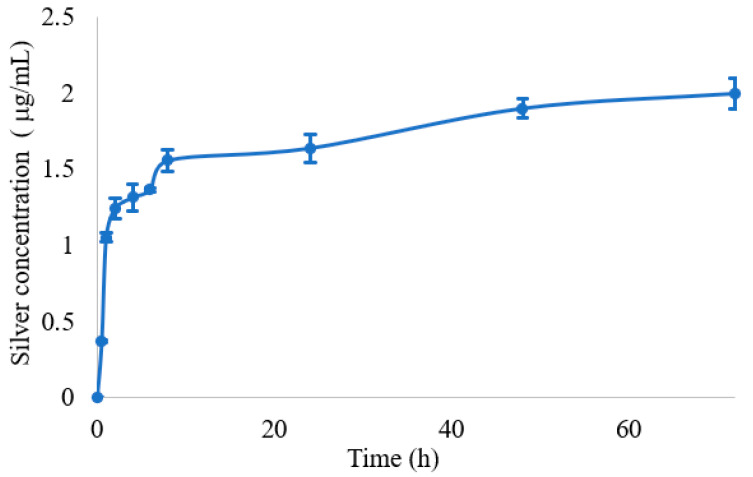
Silver release from the film β2CaCl_2_ determined by ICP analysis, expressed in concentration (μg/mL) as a function of time.

The mechanism of antimicrobial action of silver nanoparticles is ascribed to many factors and is not yet completely understood. It is reported that the activity of silver nanoparticles with size under 10 nm is mainly due to the nanoparticles itself. In fact, small silver nanoparticles can act by adhering to the surface of bacteria, altering its permeability, and can penetrate inside the cell with consequence damage to different targets. For nanoparticles with larger size, the main mechanism is due to silver ions release [39]. From the above-reported characterization, nanoparticles size resulted higher than 10 nm; thus, with the aim of evaluating silver ion release from films, an in vitro release test was performed on film β2CaCl_2_, which was chosen as a model. Silver release was carried out in simulated wound fluid up to 72 h. The test could not be performed for un-gelled films, as they dissolved in the fluid. The silver release profile showed an initial burst effect, and then, a gradual and prolonged silver release was observed, and a plateau was reached after 50 h (Figure 7). The release profile can be affected by the fluid composition, which contains chloride anions, and in fact, the plateau was observed when the condition of saturation was reached (AgCl solubility 1.9 mg/L) [40]. Silver ions release is the consequence of many steps: first, in the presence of water, gradual alginate hydration occurs, and the water penetration induces silver ions solubilization from surface of metallic silver/AgCl nanoparticles, and successively, ions diffuse across the hydrated hydrogel and are released. In the case of silver metallic nanoparticles, silver release is preceded by its oxidation. Moreover, AgCl, due to its very low solubility, ensures a slow release of silver ions [12,13,18].

In order to investigate the relationship between swelling and silver release, regression analysis of silver release for 24 h and water absorption data were applied. A correlation with R = 0.93 (Figure S3) was obtained, meaning that the silver release is correlated to the water adsorption, but other factors also come into play. Among the main models used to describe a drug-release profile, the one that best describes the release of silver from film β2CaCl_2_, was the Korsmeyer–Peppas model [41]. It is described by the following equation: Q_t_/Q_0_ = kt^n^, where Q_t_ is the drug amount released at the time t, Q_0_ is the drug amount at the beginning, and n is the diffusional release exponent. A correlation coefficient of 0.98 was obtained for *n* = 0.1, elaborating the release data in the time range of 1–48 h (Figure S3).

### 3.5. Cytotoxicity Evaluation

**Figure 8:**
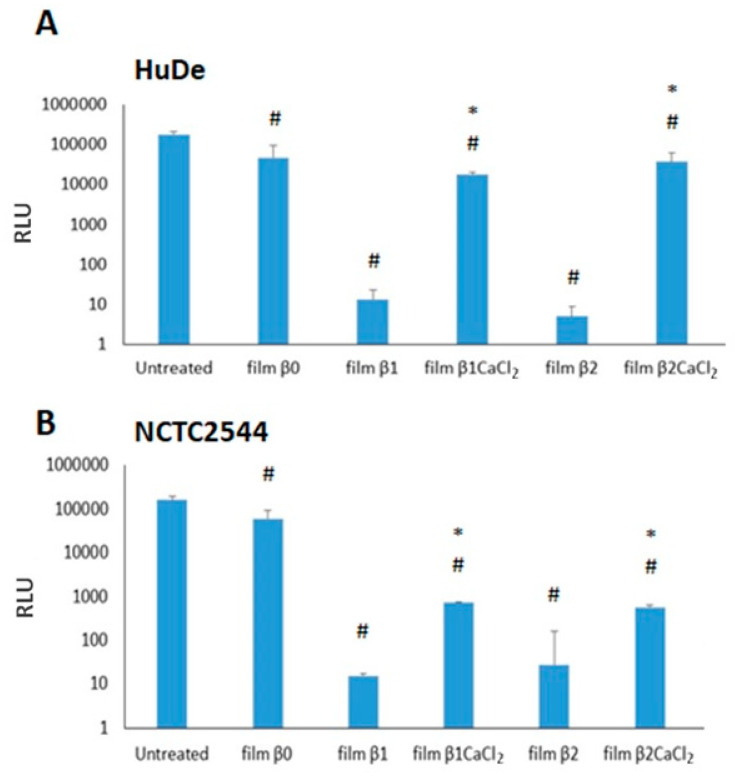
Cytotoxicity of films on HuDe (**A**) and NCTC2544 (**B**). Results are expressed as medium ± SD of RLU (relative light units). # *p* < 0.05 (cells treated with films versus untreated cells). * *p* < 0.05, (cells treated with gelled films versus cells incubated with non-gelled films).

The evaluation of the film cytotoxicity was performed by testing the viability of two human dermal fibroblast (HuDe) and keratinocyte (NCTC2544) cell lines for 24 h. Results are reported in Figure 8. The un-gelled films were toxic in both cell lines. Unlike this, film β1CaCl_2_ and film β2CaCl_2_ were always significantly less toxic than the corresponding films containing silver nitrate. In particular, film β1CaCl_2_ and film β2CaCl_2_ showed very low cytotoxicity towards fibroblasts. The low cytotoxicity of films is a remarkable result, as the use of silver nanoparticles is limited by the narrow window between silver ions and silver nanoparticles as concerns cytotoxicity against different kinds of eukaryotic cells and bacteria [42]. Cytotoxicity mechanisms of silver nanoparticles both against eukaryotic cells and bacteria or fungi is due to many factors, and among them is silver ions release, which depends on many parameters such as size and shape of nanoparticles. Moreover, the ions present in the surrounding environment can also affect the ion release. Other mechanisms of cytotoxicity are the generation of ROS and free radicals [43]. It can be hypothesized that un-gelled films, which rapidly solve in presence of water, induce a toxic silver concentration, whereas alginate gelation decreases the polymer solubility and thus stabilizes the films, preventing the rapid achievement of high silver concentrations. As concerns the use of silver or AgCl nanoparticles as antimicrobial agents in wound dressings, no clear conclusions can be drawn. Tran et al. [16] observed that composites containing AgCl nanoparticles showed to possess less antimicrobial activity than composites containing Ag^0^ nanoparticles but resulted as more cytotoxic against fibroblasts and keratinocytes. In other papers [10,11,12,14], Ag/AgCl nanoparticles are described, and Kubasceva [11] observed that hybrid (AgCl/Ag)nanoparticle/diatomite composites with a dominant content of AgCl nanoparticles showed higher antimicrobial activity, but no cytotoxicity studies were performed. Boccalon et al. [14] reported that the susceptibility of the bacterial strains toward Ag nanoparticles is lower than that of Ag/AgCl. Vosmanska et al. [44] prepared AgCl nanoparticles on cellulose dressing, which revealed the same antimicrobial activity against both *Staphylococcus epidermidis* and *Escherichia coli,* but in this paper also, no cytotoxic activity was investigated. Finally, Zhou et al. [19] obtained Ag/AgCl nanoparticles coated on graphene with high antimicrobial activity and attributed the good antimicrobial performance to the oxidative radical generation by the Ag/AgCl-graphene. The main problem in drawing clear conclusions on the different effects of Ag^0^, AgCl, and Ag/AgCl nanoparticles is due to the differences of the papers as concerns the nanoparticles’ nature, their size and shape, the way of reporting antimicrobial and cytotoxic results (for example, MIC or zone of inhibition for antimicrobial activity), and the used bacteria strains, as just previously referred [9].

## 4. Conclusions

Alginate films containing Ag/AgCl nanoparticles were obtained according to a sustainable procedure that does not involve the use of organic solvent and additional reagents, such as capping agents, as alginate itself performs this function. The immersion of previously prepared AgNO_3_ alginate films in a CaCl_2_ solution induces in one step both the alginate gelation and the AgCl nanoparticles formation. Films containing AgCl nanoparticles uniformly distributed were obtained. Moreover, the presence of metallic Ag was detected as well, due to the reducing action of alginate. The composite films showed good water absorption capacity, good antimicrobial and antibiofilm activities towards the tested microorganisms, and low toxicity for keratinocytes and fibroblasts. Un-gelled alginate films containing silver nitrate, which were prepared for comparison, showed higher cytotoxicity against both tested human cells. The lower cytotoxicity could be due to the prolonged silver ion release from Ag/AgCl nanocomposites films, which prevents the achievement of cytotoxic silver concentrations in the surrounding environment.

Based on these results, the proposed procedure represents a sustainable and successful approach for obtaining alginate Ag/AgCl nanoparticles composite films able to prevent microbial colonization and biofilm formation with reduced cytotoxicity for the tissue cells.

## Figures and Tables

**Table 1:** Film denomination and AgNO_3_ composition for film preparation.

Film	AgNO_3_ (g/100 g of Dispersion)	Gelation *	Film Thickness (μm)
β0	-	-	11 ± 1.0
β1	0.3	-	6.0 ± 1
β2	0.45	-	6 ± 1
β0CaCl_2_	-	+	19 ± 2
β1CaCl_2_	0.3	+	9 ± 2
β2CaCl_2_	0.45	+	9 ± 2

* Films β0, β1, and β2 are not gelled films. They were successively gelled to give films β0CaCl_2_, β1CaCl_2_, and β2CaCl_2_, respectively.

**Table 2:** Inhibition halos of antimicrobial films.

Inhibition Halos (mm) *				
Composite Films	*P. aeruginosa*	*S. aureus*	*S. epidermidis*	*C. albicans*
β1CaCl_2_	12.7 ± 0.6	12.0 ± 1.0	7.1 ± 0.1	8.0 ± 0.0
β2CaCl_2_	13.0 ± 0.0	12.3 ± 0.6	7.1 ± 0.0	9.0 ± 0.0
Positive control	20.0 ± 0.0	14.3 ± 4.9	14.0 ± 2.6	14.3 ± 2.1

* The results are expressed in mm and represent the mean ± S.D. of three distinct measurements. Gentamicin was the positive control for Gram-positive and Gram-negative bacteria, and fluconazole was the positive control for *C. albicans.*

## Supplementary Materials

The following supporting information can be downloaded at: https://www.mdpi.com/article/10.3390/jfb14020084/s1. Figure S1: FE-SEM images of (A) film β1 immersed in 0.8% CaCl_2_ solution, (B) film β1 immersed in 1.2% CaCl_2_ solution, (C) film β2 immersed in 0.8% CaCl_2_ solution, (D) film β1 immersed in 1.2% CaCl_2_ solution; Figure S2: FE-SEM images of film β0CaCl_2_; Figure S3: Regression analysis of cumulative silver release (%) from film β2CaCl_2_ against its water hydration data (percentage) for 24 h (left) and in vitro silver ions release from film β2CaCl_2_ as a function of time^0.1^ according to Korsmeyer–Peppas equation (right).
