# Supplementary material for: Alginate Ag/AgCl Nanoparticles Composite Films for Wound Dressings with Antibiofilm and Antimicrobial Activities
Source: J Funct Biomater. 2023 Feb 1;14(2):84. doi: 10.3390/jfb14020084 (PMC9968148; doi:10.3390/jfb14020084)
Supplement: Supplementary file 1 [file jfb-14-00084-s001.zip › jfb-2144942-supplementary.pdf]

Alginate Ag/AgCl nanoparticles composite films for wound dressings with antibiofilm and antimicrobial activities

Matteo Puccetti[a], Anna Donnadio[a], Maurizio Ricci[a], Loredana Latterini[b], Giulia Quaglia[b], Donatella Pietrella[c], Alessandro Di Michele[d], Valeria Ambrogi[a]*

[a] Dipartimento di Scienze Farmaceutiche, Università degli Studi di Perugia, Via del Liceo 1, 06123 Perugia, Italy

[b] Nano4Light Lab, Dipartimento di Chimica, Biologia e Biotecnologie, Università degli Studi di Perugia, Via Elce di Sotto, 8, 06123 Perugia, Italy

[c] Dipartimento di Medicina e Chirurgia, Università degli Studi di Perugia, Via Piazzale Gambuli, 1, 06129 Perugia, Italy

[d] Dipartimento di Fisica e Geologia, Università degli Studi di Perugia, Via Pascoli, 06123 Perugia, Italy

* Corresponding Author:

Valeria Ambrogi, e-mail address: valeria.ambrogi@unipg.it Phone:+390755855125.

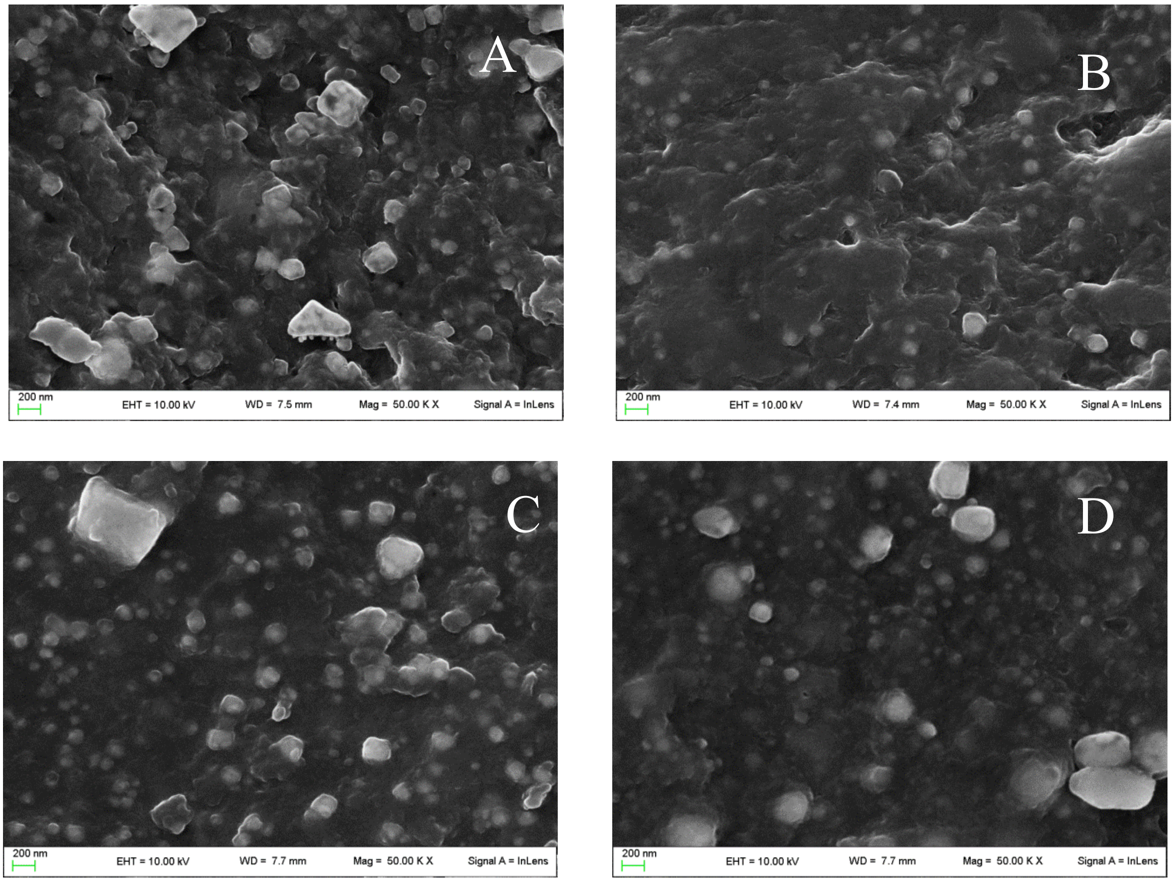

Figure S1: FE-SEM images of (A) filmβ1immersed in 0.8% $CaCl_2$ solution, (B) film β1immersed in 1.2% $CaCl_2$ solution, (C) film β2 immersed in 0.8% $CaCl_2$ solution, (D) film β1 immersed in 1.2% $CaCl_2$ solution.

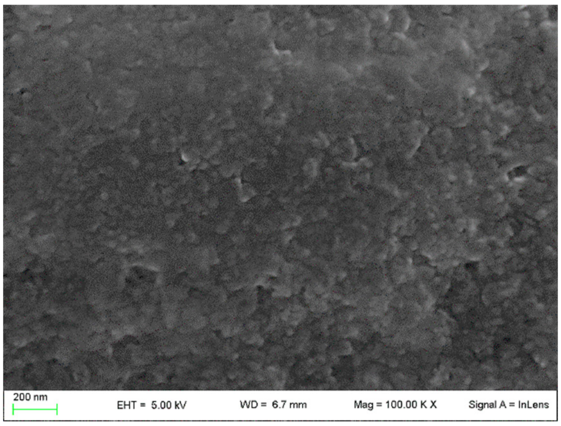

Figure S2: FE-SEM images of film $\beta 0CaCl_2$.

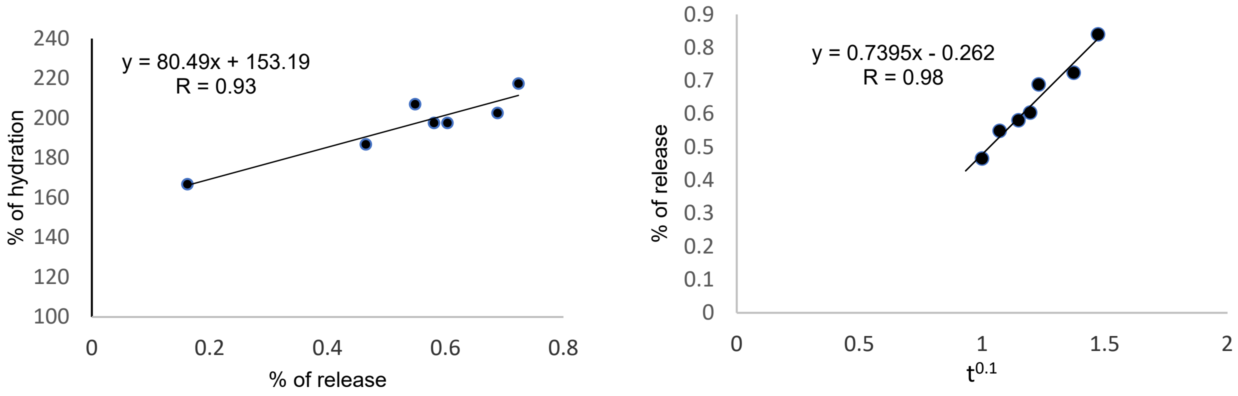

Figure S3: Regression analysis of cumulative silver release (%) from film β2CaCl₂ against its water hydration data (percentage) for 24 h (left) and in vitro silver ions release from film β2CaCl₂ as a function of time$^{0.1}$ according to Korsmeyer-Peppas equation (right).
